# Analysis of Leaf and Soil Nutrients, Microorganisms and Metabolome in the Growth Period of *Idesia polycarpa* Maxim

**DOI:** 10.3390/microorganisms12040746

**Published:** 2024-04-07

**Authors:** Tao Zhang, Shasha Wang, Sohel Rana, Yanmei Wang, Zhen Liu, Qifei Cai, Xiaodong Geng, Qiupeng Yuan, Yi Yang, Chao Miao, Xiaoyan Xue, Li Dai, Zhi Li

**Affiliations:** College of Forestry, Henan Agricultural University, Zhengzhou 450046, Chinasohel.sam@live.com (S.R.);

**Keywords:** *Idesia polycarpa*, rhizosphere soil, endophytes, metabolites

## Abstract

*Idesia polycarpa* Maxim is an emerging oil plant species. Understanding its microecological characteristics and internal mechanisms can serve as a basis for field management and the screening and application of growth-promoting bacteria during the growth phase of young trees. This study used three-year-old young *I. polycarpa* to analyze the tree’s root morphology, soil, and leaf nutrient status variations from May to October. In addition, differences in the rhizosphere soil, leaf metabolites, and microorganisms were observed. The results showed that, from May to October, the total nitrogen (TN) in the soil significantly decreased, whereas the TN, total potassium (TK), and total phosphorus (TP) in the leaves differed (*p* < 0.05). The leaf-dominant bacteria changed from Pseudomonadota to Firmicutes phylum. In addition, the relative abundance of soil and leaf-dominant bacteria decreased. The study found that the soil and leaf differential metabolites were mainly sugars and phenolic acids. The soil bacterial community showed a significant correlation with soil pH, available potassium (AK), available phosphorus (AP), and TN (*p* < 0.05). Further, the soil fungal community was significantly correlated with pH and AK (*p* < 0.001). TP, pH, and TK were the main factors influencing the leaf bacterial community, while the leaf fungal community was significantly correlated with five factors, including pH, TC, and TN. The root morphology was also mainly affected by pH, *Pedomicrobium* sp., *Talaromyces* sp., *Penicillium* sp., and D-Mannitol 2.

## 1. Introduction

*Idesia polycarpa* is a deciduous tree of the *Idesia* genus in Salicaceae. Its tall stature, straight trunk, and gorgeous fruit make it popular for landscapes, gardens, and restoration projects [1]. The fruit has a high oil content, and the content of unsaturated fatty acids in the oil is as high as 77%, of which the linoleic acid content is as high as 62.9% [2]. It is a woody plant species with a high development value. Young trees are relatively fragile throughout life, and their growth and development are closely related to their microhabitat. A young tree’s survival determines the population’s continuation [3]. It exhibits different adaptation strategies during growth. Due to different adaptation strategies, the soil–leaf microecology will change. In studying the root growth status of young trees, we can better understand their adaptation to different environments and analyze the factors that affect their growth [4].

Nutrients are essential for seedlings, forest growth, and woodland productivity [5]. Roots are the main organs that allow plants to absorb nutrients, such as nitrogen (N), phosphorus (P), and potassium (K), and are the basis for plant growth and development. Young trees show significant differences in nutrient utilization during their growth [6]. Zhang’s [7] research found that apple trees absorb less N in the early stage of the growth period, and with the progress of growth, the N absorption capacity of young trees gradually increases. In addition, studies found that young beech trees’ leaf numbers and leaf areas were inhibited differently under different N and P nutrient conditions [8]. A phosphorus deficiency inhibits the growth of *Palamicella belloni* seeding and reduces nutrient uptake efficiency [9]. In addition, it has been found that young broadleaves are more affected by soil nutrients than adult trees [10]. However, a positive correlation exists between young tree growth and leaf phosphorus content in the central Amazon [11]. Other studies on young oak trees found that, with the progress of the growth period, the content of N and K in the leaves decreased significantly [12]. Therefore, clarifying the changes in nutrients at different growth stages for forest land management is significant. In addition, there are many microbial communities in leaves and soil, which also exhibit different properties at different times.

Another study [13] reported that the relative abundance of dominant bacteria in different leaf periods differed. The dominant bacteria genus was Edible Monocytes in young leaves, and the dominant endophytic bacteria in mature leaves was Pseudomonas. The metabolic pathways related to amino acids and total soluble sugars changed with the development period of flowers [14]. Lusi et al. [15] used the high-throughput sequencing method to analyze the relationship between bacterial and fungal diversity and root traits in the rhizosphere soil of temperate mountain forests. Furthermore, Xiao [16] found eight microbial communities dominated by *Acidocella aluminiidurans*, *Tepidisphaera mucosa*, and *Vibrio bacteriovorus* bacteriophage during rhododendron development to be significantly correlated with metabolite cluster groups composed of quercetin-5-O-glucuronide, isorhamnetin-3-O-(6″-malonyl) glucoside, and Vitexilactone. This suggests that the inter-root bacterial community is closely linked to metabolites. The soil–leaf microecological characteristics and internal mechanism of *I. polycarpa* are related to growth and development, and finding the connection between them may be beneficial to its development and utilization [17]. 

Previous studies on *I. polycarpa* have focused on adult plants [18,19,20], and they rarely involved young trees. In particular, there are relatively few studies on the microecology of the underground and above-ground areas of young trees, or only one part of the soil or leaves was involved in these studies. There has been no specific classification study on the soil and leaf microecology of young trees. In this study, young *I. polycarpa* trees were used as experimental materials to observe the monthly changes in root system traits, the rhizosphere soil and leaf nutrients during May and October, and the changes in the rhizosphere soil and leaf metabolites and microbial communities in the two periods. On this basis, the differences in each period of the young trees’ development and the relationship between microorganisms, nutrients, and metabolites were analyzed. In addition, the internal mechanism of the soil and leaf microecology of the young trees in different growth periods was elucidated. The study data build a solid foundation for cultivating and managing young *Idesia polycarpa* trees.

## 2. Materials and Methods

### 2.1. Study Site

The experimental site is located at the Forestry Experimental Station (112°42′114°14′ E, and 34°16′34°58′ N) of Henan Agricultural University, Zhengzhou, China. It belongs to a temperate continental monsoon climate, and the average annual temperature is 14.2 °C. The extreme high temperature is 43 °C, and the low temperature is −17.9 °C. The annual precipitation is 650.1 mm, the annual sunshine is 2400 h, and the frost-free period is 215 d. The soil type of the experimental plot is sandy loam [21].

### 2.2. Experimental Material

The experimental materials were young 3-year-old *I. polycarpa* trees planted in the test site with a spacing of 1.5 m × 1.5 m. The bud swelling period began on 15 March, the bud break period began on 20 March, the leaf spreading period began in early April, and the leaf falling period began in September [22,23,24]. For sampling, six plants with robust growth and no obvious pests or diseases were randomly selected from the test plot, and the surrounding environment was maintained regularly.

### 2.3. Observation of Root Morphology Indicators

In December 2021, colorless and transparent micro-root tubes 100 cm in length were buried around the plants at an inclined angle of 45° to the ground at a vertical depth of about 50 cm. The underground gaps along the root tubes were filled with diluted slurry, the exposed portion of the ground was sealed and wrapped in a black film for shading, and the tube was inserted in the north–south direction of each plant. The root canal scans were performed six times from May to October 2022 using the plant root growth monitoring system CI-600 (CID Bio-Science, Camas, WA, USA) and WINRHIZO TRON (Regent Instruments Inc., Québec City, QC, Canada). The study measured the total root volume, root length, number of root tips, total average root diameter, and total root surface area of the root system. The root morphology correlation data (soil nutrients, microorganisms, and metabolome) were analyzed using R software (V4.1.0), and redundancy analysis (RDA) was carried out and correlation heat maps were drawn.

### 2.4. Soil and Leaf Sample Collection and Determination of Physical and Chemical Properties

Soil sample collection: A sterilized root drill was used to drill 0–20 cm soil samples in the four directions of the selected plant, and the distance was 20 cm from the tree trunk. After mixing evenly, the samples were placed in an incubator with dry ice and returned quickly to the laboratory. Subsequently, one part of the soil sample was placed in a refrigerator at −80 °C to determine soil microorganisms and metabolites, and the other part was dried in the laboratory to determine soil nutrient content.

Leaf sample collection: In each sampling tree (the same tree as the soil sample) in the periphery of the canopy in the southeast, northeast, and northwest directions, we picked the top of the branch tips of the plant leaves at a height of 1.5–2 m, we collected 20 pieces of leaves from each plant, and repeated this three times. The collected leaf samples were put into a sealed bag, kept in a numbered place, and put in an incubator with dry ice to be returned to the laboratory in time. The collected leaf samples were quickly washed with 0.1% neutral detergent on both sides, washed three times with tap water, and soaked in 0.2% HCL for 30 s. Subsequently, they were washed three times with tap water, and finally, they were rinsed three times with distilled water. The cleaned leaf samples were divided into three parts, one of which was dried at 55 °C after baking at 105 °C for 30 min until the mass was constant, and then ground, sieved, and stored in a sealed bag for the determination of physicochemical properties of the leaves. The other parts were placed in a refrigerator at −80 °C to determine the leaves’ microorganisms and metabolome.

Determination of soil pH: 10 g (accurate to 0.01 g) of soil sample was weighed and put in 50 mL of boiling water; 25 mL of CO_2_-free water was added (soil–liquid ratio 1:2.5) [25], and the mixture was stirred with a glass rod for 1 min to fully disperse the soil particles, and then after 30 min the pH was measured with an acidimeter (LC-PH-3S, Shanghai LiChen Bangxi Instrument Equipment Co., Shanghai, China). The pH value of the upper layer of the sample was measured, and each measurement was repeated three times to obtain the soil pH value. 

Determination of leaf pH: 0.3 g (to 0.5 g) of the sample was weighed using an analytical balance (FA2004, Shanghai Shangping Instrument Co., Ltd., Shanghai China), placed in a 10 mL centrifuge tube, and 3 mL of CO_2_-free purified water was added (leaf–liquid ratio of 1:10 [26]). A vortex mixer (1592D, Thermo Fisher Scientific, Waltham, MA, USA) was used to mix the sample with the purified water, and then the centrifuge tube containing the sample was placed in a constant-temperature shaker at 25 °C for 60 min. The centrifuge tube containing the sample was placed in a 25 °C thermostatic shaker for 60 min. After the shaker was stopped, the tubes were centrifuged in a centrifuge for 2 min, and the samples were analyzed using an acidimeter (LC-PH-3S, Shanghai LiChen Bangxi Instrument Equipment Co., Shanghai, China) The pH of the supernatant was measured using an acid meter (LC-PH-3S, Shanghai LiChen Bangxi Instrument Equipment Co., Ltd., Shanghai, China).

Soil alkaline nitrogen (AN) was determined by the alkaline diffusion method—the soil samples were treated with 1. 8 M NaOH and Zn-FeSO_4_ reductant and titrated with 0.1 M hydrochloric acid standard solution [27]. In our study, the total carbon (TC) and total nitrogen (TN) of the soil and leaves were determined using an automated elemental analyzer (Euro Vector EA3000, Shanghai Wolong Instrument Co., Ltd., Shanghai, China). The soil’s available phosphorus (AP) was determined using 0.5 M NaHCO_3_ leaching, molybdenum antimony resistance, and the colorimetric method. The soil’s available potassium (AK) was determined by 0.5 M NaHCO_3_ leaching using the flame photometer method. Soil and leaf total phosphorus (TP) was determined using H_2_SO_4_-H_2_O_2_ decoction and the molybdenum antimony resistance colorimetric method. In addition, soil and leaf total potassium (TK) was determined by the H_2_SO_4_-H_2_O_2_ decoction flame photometer method. The data were analyzed by ANOVA using IBM SPSS v. 26 (IBM Corp., Armonk, NY, USA, https://www.ibm.com/, accessed on 30 November 2022), and Origin 2017 (www.OriginLab.com, accessed on 30 November 2022) was used to draw histograms of nutrient contents. 

### 2.5. Determination of Leaf and Soil Microorganisms

#### 2.5.1. DNA Extraction, PCR Amplification, and Library Preparation

Total DNA was extracted from leaves and soil according to the instructions of the E.Z.N.A.^®^ soil kit (Omega Bio-tek, Norcross, GA, USA), and DNA concentration and purity were assayed using the NanoDrop2000 (Thermo Fisher Scientific, USA), and the quality of the total DNA extraction was assayed using 1% agarose gel electrophoresis. The upstream primer 338F (5′ACTCCTACGGGGAGGCAGCAG-3′) and downstream primer 806R (5′-GGACTACHVGGGTWTCTAAT-3′) were used to analyze the bacterial 16S rRNA gene V3-V4 region. The upstream primer ITS5-1737F (50-GGAAGTAAAAGTCGTAACAAGG-30) and downstream primer ITS2-2043R (50-GCTGCGTTCTTCATCGATGC-30) were used to amplify the ITS1-ITS2 region of the fungus. The amplification system was 20 μL, 4 μL 5 × FastPfu buffer, 2μL 2.5mM dNTPs, 0.8 μL primer (5 μm), 0.4 μL FastPfu polymerase; 10 ng DNA template. The amplification conditions were 95 °C pre-denaturation for 3 min, 27 cycles (95 °C denaturation for 30 s, 55 °C annealing for 30 s, 72 °C extension for 30 s), and finally 72 °C for 10 min (PCR instrument: ABI GeneAmp^®^ 9700). PCR products were recovered using 2% agarose gel, purified using AxyPrep DNA Gel Extraction Kit (Axygen Biosciences, Union City, CA, USA), eluted with Tris-HCl, and detected by 2% agarose electrophoresis. Quantification was performed using QuantiFluor™-ST (Promega, Madison, WI, USA). The three replicates of soil and leaf samples were set up in May and October, and the amplified products were sequenced using the Illumina MiSeq platform (Illumina, San Diego, CA, USA) for paired-end sequencing of community DNA fragments. Sequencing was performed by Parsonage (Shanghai, China).

#### 2.5.2. Bioinformatics Analysis

The analysis followed the “Atacama soil microbiome tutorial” of QIIME2 docs and customized program scripts (https://docs.qiime2.org/2019.1/, accessed on 30 November 2022). Briefly, raw data FASTQ files were imported into the format that could be operated by the QIIME2 system using the QIIME tools import program. Demultiplexed sequences from each sample were quality filtered and trimmed, de-noised, and merged, and then the chimeric sequences were identified and removed using the QIIME2 dada2 plugin to obtain the feature table of amplicon sequence variant (ASV) [28]. The QIIME2 feature-classifier plugin generated the taxonomy table to align ASV sequences with a pre-trained GREENGENES 13_8 99% database. Any contaminating mitochondrial and chloroplast sequences were filtered using the QIIME2 feature-table plugin. Bacteria and fungi were annotated to species level using the database, respectively, and, based on the species annotation information, OTUs (Operational Taxonomic Unit) and their contained sequences were annotated to chloroplasts and mitochondria, and those that could not be annotated to the kingdom level were removed. The QIIME2 software (V 2022.11) was used to visualize the compositional distribution of the samples at phylum and genus levels and present the microbial communities in terms of relative abundance. The R software was used to plot histograms at the phyla level and clustered plots at the genus level with relative abundance, perform correlation analyses between the microbial communities and the relevant nutrient factors, and redundancy analysis (RDA, explain the relationship between two variables) plots and correlation heat maps were drawn.

### 2.6. Metabolome Analysis

#### 2.6.1. Metabolite Extraction

Soil sample extraction: The soil sample was ground to a powder, and a 0.5 g soil sample was weighed and added the methanol–isopropanol–water (3:3:2) extraction solution, and shaken well at room temperature for 3 min before being added to an ice water bath and sonicated for 20 min. Then, the supernatant was centrifuged, added to the internal standard (10 μg/mL), placed in the nitrogen blowing apparatus, blown dry, and then lyophilized. Then, we added 0.1 mL of pyridinium methoxide (10 μg/mL), oximized it for 2 h in the 37 °C oven, and then added 0.1 mL of BSTFA (with 1% TMCS), and freeze-dried it at 37 °C in an oven. After oximization for 2 h, we took 0.1 mL of BSTFA (with 1% TMCS) and reacted it for 30 min in the oven at 37 °C to obtain the derivatization solution; this was then diluted to 1 mL, filtered, and stored in the refrigerator at −20 °C. The soil and leaf samples were prepared with three sample replicates each in May and October.

Leaf sample extraction: Leaf samples were ground to powder, and 100 mg of this powder was weighed, dissolved in 1.2 mL of methanol extract, vortexed once every 30 min for 30 s, and vortexed a total of 6 times, and at the end the samples were placed in the refrigerator at 4 °C overnight. We centrifuged (speed 12,000 rpm, 10 min) the supernatant, filtered it, and then it put into a test tube for preservation. The soil and leaf samples were prepared with three sample replicates each in May and October.

#### 2.6.2. The UPLC-MS/MS Analysis

A gas chromatography–tandem mass spectrometer (GC-MS) instrument from Aglient 8890-5977B was used to collect soil metabolites, and the metabolites of soil samples were analyzed by mass spectrometry based on the S_TMS_MWGC database. Based on the ultra-performance liquid chromatography–tandem mass spectrometry (UPLC-MS/MS) detection platform and the MWDB database, the leaf sample metabolites were collected, and substances were characterized by using the VIP value (Variable Importance in Projection) from the R software multiplicity of variance OPLS-DA model for differential metabolite analysis. The conditions of FC ≥ 2 or FC ≤ 0.5 and VIP ≥ 1 were selected to screen the metabolites that caused significant differences between the two groups of samples, and the top 10 substances that had upward and downward variations of differential metabolites in the two groups were selected to plot the bar graphs. The substances in the two groups of samples were used to draw the bar graph and KEGG pathway analysis was performed according to the results of differential metabolites. We used R software to analyze the correlation of metabolite microorganisms and draw the correlation heat map.

## 3. Results

### 3.1. Characteristics of Fine Roots

**Figure 1:**
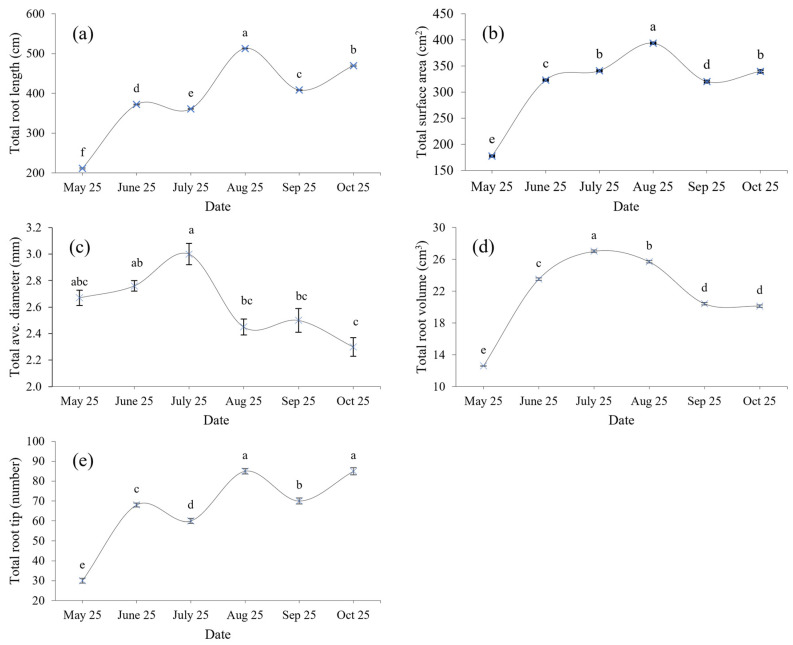
Differences in root characteristics of young trees in different periods. The lowercase letters indicate significant differences in root morphology at different stages (*p* < 0.05). Data values represent the mean ± SE. (**a**) Total Root Length; (**b**) Total Surface Area; (**c**) Total Average Diameter; (**d**) Total Root Volume. (**e**) Total Root Tip.

There are significant differences in the morphological characteristics of the fine roots (Figure 1a–e). The total root length, surface area, volume, and apex number of fine roots in October were higher than in May by 122%, 91%, 60%, and 183%, respectively. The total root length and number root tips of saplings showed a fluctuating growth trend with time. The average root diameter and total volume showed a trend of increasing first and then decreasing, and the total root surface area increased.

### 3.2. Soil Physical and Chemical Properties Analysis

**Figure 2:**
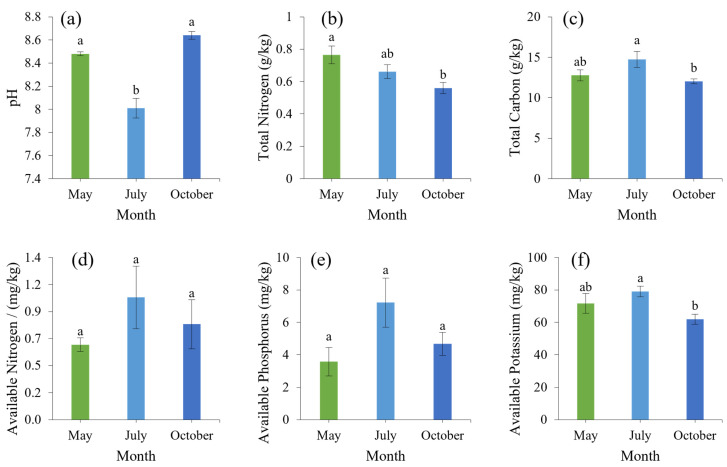
The physicochemical properties of soil of young trees. The lowercase letters indicate significant differences in root morphology at different stages (*p* < 0.05). Data values represent the mean ± SE. (**a**) pH; (**b**) Total Nitrogen; (**c**) Total Carbon; (**d**) Available Nitrogen; (**e**) Available Phosphorus; (**f**) Available Potassium.

The pH value of each soil decreased first and then increased during the whole growing period (Figure 2a). The total nitrogen content of the soil decreased gradually with the change in the growth period, and the total nitrogen content of young trees in October was significantly lower than that in May, by 33%. The total carbon, available nitrogen, phosphorus, and potassium showed a trend of increasing first and then decreasing with the change in time (Figure 2b–f). The soil’s total carbon content was significantly higher in July than in October, by 23%, and soil’s AK content was significantly higher in July than in October, by 28%. There was no significant difference in the soil’s available nitrogen and potassium content throughout the period.

### 3.3. Leaves Physical and Chemical Properties Analysis

**Figure 3:**
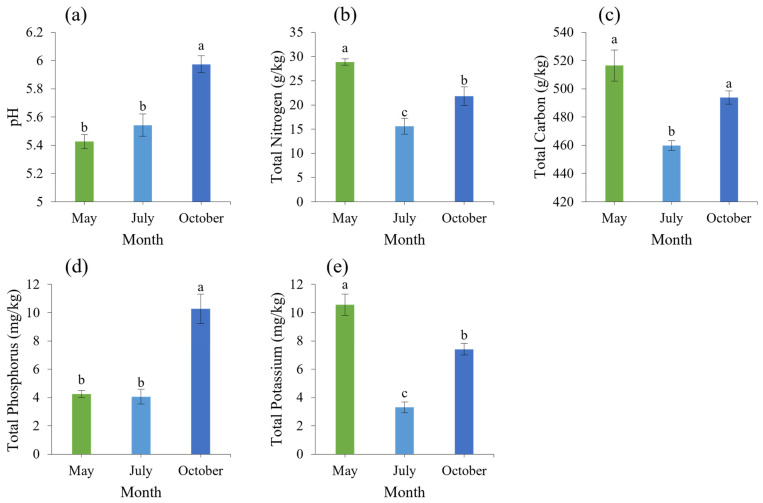
The physicochemical properties of leaves of young trees. The lowercase letters indicate significant differences in root morphology at different stages (*p* < 0.05). Data values represent the mean ± SE. (**a**) pH; (**b**) Total Nitrogen; (**c**) Total Carbon; (**d**) Total Phosphorus; (**e**) Total Potassium.

The pH value of leaves (Figure 3a) increased gradually throughout the growth period, and the pH value was acidic throughout the whole period, which was significantly, 11%, higher in October than in May and July. The contents of leaf total nitrogen, total carbon, total phosphorus, and total potassium decreased first and then increased with the change in growth period (Figure 3b–e). Among them, the total nitrogen and potassium levels in May were significantly higher than those in October and July, and total carbon in May and October was 32.18% and 42.59% higher than in July.

### 3.4. Analysis of Soil Microbial Diversity in the Rhizosphere

#### 3.4.1. Analysis of Soil Bacterial Diversity in the Rhizosphere

**Figure 4:**
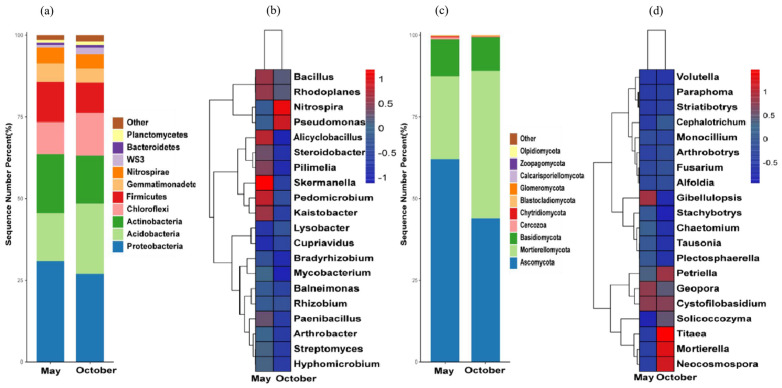
Soil bacterial diversity in different periods. (**a**) Histogram of horizontal phyla distribution of bacteria; (**b**) heat map of dominant bacteria genera. Diversity of soil fungi in different periods (red indicates a positive correlation, blue indicates a negative correlation, and 0.5 indicates a significant correlation). (**c**) Histogram of horizontal phyla distribution of fungi; (**d**) heat map of dominant fungi genera. Red indicates a positive correlation, blue indicates a negative correlation, and 0.5 indicates a significant correlation.

The bacterial OTU sequences obtained from the May and October soil samples were classified and annotated and were divided into 44 phyla and 250 genera. At the phyla level, soil bacteria were mainly concentrated in Pseudomonadota (23.61~39.70%), Acidobacteriota (9.36~22.86%), Actinobacteriota (13.02~22.29%), Chloroflexota (7.14~14.24%), Bacillota (9.87~17.03%), Gemmatimonadota (3.29~6.49%), and Nitrospinota (4.23~5.24%), while WS3 (0.45~1.85%), Bacteroidota (0.49~2.89%) and Planctomyceota (0.23~0.88%) accounted for less (Figure 4a).

In the soil samples from May, the genera with higher relative abundances mainly included *Bacillus*, *Rhodoplanes*, *Skermanella*, *Alicyclobacillus*, *Pedomicrobium,* and *Kaistobacter*; in the soil samples from October, the genera with higher relative abundances were *Nitrospira* and *Pseudomonas* (Figure 4b).

#### 3.4.2. Analysis of Soil Fungal Diversity of Rhizosphere

The soil fungi in the rhizosphere of the young trees belonged to five phyla and thirty-eight genera. The soil fungal communities that accounted for the largest proportion at the phyla level included Ascomycota (36.96~71.32%), followed by Mortierellomycota (36.96~71.32%) and Basidiomycota (7.56~10.26%), and the rest were lower in abundance (Figure 4c).

The composition of the fungal community in the rhizosphere soil samples of each group is significantly different at the genus level. In the soil sample in May, *Gibellulopsis* and *Geopora* were the dominant genera in the soil fungal community of young trees; in the soil sample in October, the dominant genera in the young tree soil were *Petriella*, *Titaea*, *Mortierella*, and *Neocosmopora* (Figure 4d).

### 3.5. Analysis of Leaf Microbial Diversity

#### 3.5.1. Analysis of Leaf Bacteria Diversity

**Figure 5:**
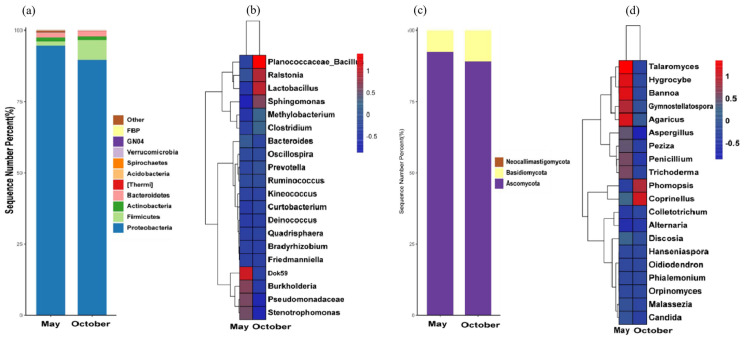
Diversity of leaf bacteria in different periods. (**a**) Histogram of phyla horizontal distribution of endophytic bacteria; (**b**) heat map of dominant endophytic bacteria genera. Diversity of leaf fungi in different periods (red indicates a positive correlation, blue indicates a negative correlation, and 0.5 indicates a significant correlation). (**c**) Histogram of phyla horizontal distribution of endophytic fungi; (**d**) heat map of dominant endophytic fungi genera. Red indicates a positive correlation, blue indicates a negative correlation, and 0.5 indicates a significant correlation.

The young trees had 30 phyla and 259 genera of leaf bacteria. There were four bacterial phyla with an average relative abundance >1% at the phyla level, namely Proteobacteria (79.88~97.76%), Firmicutes (1.04~14.73%), Actinobacteria (0.41~4.37%), and Bacteroidetes (0.44~3.67%); the relative abundance of Firmicutes was significantly higher in October than in May (Figure 5a).

At the genus level, the abundance of Dok59 in young tree leaves was higher in the May leaf sample, followed by Burkholderia, Pseudomonas, and Stenotrophomonas. In the October leaf sample, the genera with the higher relative abundance in the young trees’ leaves included *Bacillus*, *Lactobacillus*, *Ralstonia*, and *Sphongomonas* (Figure 5b).

#### 3.5.2. Analysis of Leaf Fungal Diversity

The endophytic fungi in the leaves of the young trees belonged to three phyla and twenty-eight genera. At the phyla level, the endophytic fungal community with the largest proportion was Ascomycota (87.44~92.75%), followed by Basidiomycota (7.25~12.56%) (Figure 5c). The genera with a relatively high abundance in the leaves of young trees at the genus level were mainly Talaromyes, Hygrocybe, Bannoa, Agaricus, and Gymnostellatospora in the May leaf sample. In the October leaf sample, the genera with a relatively high leaf abundance were *Coprinellus* and *Phomopsis* (Figure 5d).

### 3.6. Correlation Analysis between Microbial Community and Nutrient Factors

**Figure 6:**
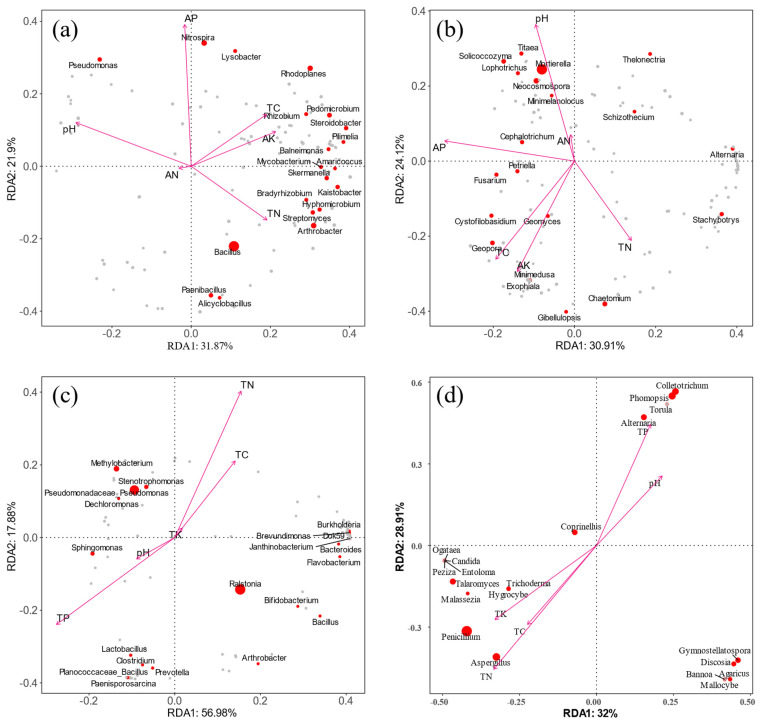
Redundancy of soil microorganisms and soil nutrient factors (**a**) bacteria, (**b**) fungi. The redundancy of leaf microorganisms and leaf nutrient factors (**c**) bacteria, (**d**) fungi.

In order to understand the effect of soil nutrient factors on rhizosphere soil microorganisms, the rhizosphere soil bacterial and fungal communities in the top 20 genera according to relative abundance were used to conduct redundancy analysis with pH and five soil nutrient factors (Figure 6a,b). The results showed that pH (*p* = 0.001), available potassium (*p* = 0.002), available phosphorus (*p* = 0.002), and total nitrogen (*p* = 0.034) were the main factors influencing the soil bacterial communities. The *Imperus*, *Arthrobacter*, *Rhizobium*, *Skermanella*, and *Streptomyces* were positively correlated with the soil’s available potassium and total nitrogen. The *Alicyclobacillus* and *Mycobacterium* were positively correlated with total nitrogen. The *Mycobacterium*, *Pseudomonas*, *Copperus*, *Rhizobium*, and *Nitrospira* were positively correlated with soil pH and available phosphorus. The soil fungal community was significantly correlated with pH (*p* = 0.001) and available potassium (*p* = 0.005), and mainly *Discosia*, *Aspergillus*, *Aspergillus*, *Pseudomycetes*, and *Microclomyces* were positively correlated with pH. The *Pseudomycetes*, *Trichoderma*, and *Trichoderma* were positively correlated with total carbon, available potassium, and phosphorus. The *Penicillium*, *Gymnostellatospora*, *Mortierella*, and *Cottonia* were positively correlated with available potassium, and the dominant genus of young trees in May and October (10 genera) were positively correlated with total nitrogen.

A redundancy analysis was conducted on the dominant microbial genus and leaf nutrient factors in leaves (Figure 6c,d). The results showed that total phosphorus (*p* = 0.001), total nitrogen (*p* = 0.006), and total carbon (*p* = 0.02) significantly affected the leaf endophytic bacterial community structure. Among them, the dominant bacteria genera, *Dok59*, *Burkholderia*, *Oligomonas*, and *Pseudomonas,* in the May leaf sample were positively correlated with total potassium, total nitrogen, and total carbon. Seven of the genera, including *Bacillus*, *Lactobacillus*, and *Lauria*, were positively correlated with total phosphorus. Eight of the genera, including *Sphingomonas*, *Methylobacter*, and *Paeomonas*, were positively correlated with pH. The leaf fungal community was significantly correlated with pH (*p* = 0.001), total potassium (*p* = 0.001), total carbon (*p* = 0.001), total nitrogen (*p* = 0.001), and total phosphorus (*p* = 0.006). The main manifestations were *Basketella*, *Umbelus*, *Bannoa*, *Umbelus*, and *Gymnostellatospora*, which were positively correlated with total potassium. The *Trichoderma*, *Malassezia*, *Aspergillus*, *Tinospora*, *Phytophthora*, *Candida*, *Botularia monocytogenes*, *and Penicillium* were positively correlated with total potassium, nitrogen, and carbon. The *Phomopsis*, *Pseudomyces*, *Alternaria, Hansonia*, *Orpinomyces*, and *Discosia* were positively correlated with pH and total phosphorus; *Pseudomonas* were positively correlated with total phosphorus.

### 3.7. Metabolomic Analysis of Rhizosphere Soil and Leaves of Young Plants at Different Stages

**Figure 7:**
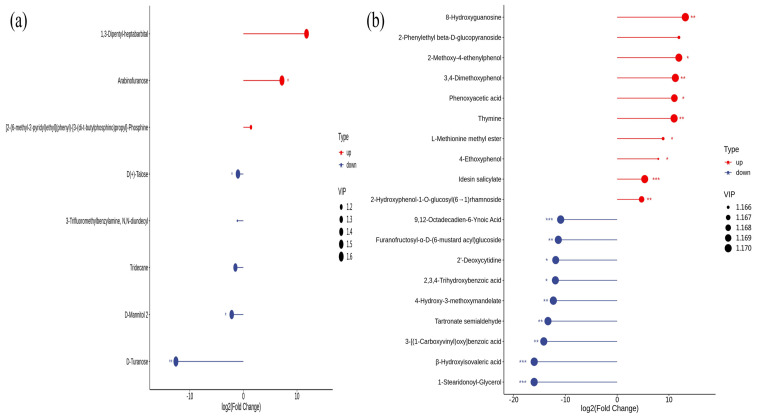
(**a**) Soil differential metabolite analysis at different periods. Up-regulated means that the expression of metabolites in October increased relative to May, and down-regulated means that the expression of 10 metabolites decreased relative to May. VIP: Variable importance in projection. (**b**) Analysis of Differential Metabolites in Leaves at Different Periods. The single asterisk (*) represents the significance of *p* < 0.05, the double asterisk (**) represents *p* < 0.01, and the triple asterisk (***) represents *p* < 0.001 value.

Differential metabolites were found in the soils in May and October. A total of eight differential metabolites were detected in the soil samples (Figure 7a), of which three metabolites were up-regulated, including a heterocyclic compound (one), a carbohydrate (one), and another class (one). Five metabolites were down-regulated, including lipids (one), ribosomes (three), and other classes (one). There were three types of metabolites with up-regulated changes, namely, heterocyclic compounds; 1, 3-Dipentyl-heptabarbital carbohydrates; and Arabinofurano heterocyclic compounds: [2-(6-methyl-2-pyridyl) ethyl] (phenyl)-[3-(di-t-butylphosphino) propyl]-Phosphine. There were three types of metabolites with significant down-regulated changes, including carbohydrates: D(+)-Talose, D-Mannitol 2, D-Turanose; others: 3-Trifluoromethylbenzylamine; and lipids: Tridecane.

Differential metabolite of leaves in May and October—A total of 302 differential metabolites were detected in the leaf samples (Figure 7b), of which 99 metabolites were up-regulated, including 49 phenolic acids, 16 lipids, 9 organic acids, 5 nucleotides and their derivatives, 10 amino acids and their derivatives, and 10 other types. In addition, 203 metabolites were down-regulated, including 68 phenolic acids, 46 lipids, 27 amino acids and their derivatives, 22 other types, 18 nucleotides and their derivatives, and 29 organic acids. The top ten metabolites with the up-regulation changes were from three categories, namely phenolic acid compounds such as 2-Phenylethyl beta-D-glucopyranoside, 2-Methoxy-4-ethenylphenol, 3,4-Dimethoxyphenol, Phenoxyacetic acid, 4-Ethoxyphenol, Idesin salicylate, 2-Hydroxyphenol-1-O-glucosyl (6→1) rhamnoside; amino acids and their derivatives L-Methionine methyl ester; and nucleotides and their derivatives 8-Hydroxyguanosine, Thymine. There were four classes from the top ten metabolites with the largest down-regulation changes, including phenolic acid compounds: Furanofructosyl-α-D-(6-mustard acyl) glucoside, 3-[(1-Carboxyvinyl) oxy] benzoic acid, 2,3,4-Trihydroxybenzoic acid, 2-Hydroxy-3-phenylpropanoic acid; nucleotides and their derivatives: 2′-Deoxycytidine; organic acids: Tartronate semialdehyde, 4-Hydroxy-3-methoxymandelate, β-Hydroxyisovaleric acid; and lipids: 12-Octadecadien-6-Ynoic Acid, 1-Stearidonoyl-Glycerol.

### 3.8. KEGG Analysis

**Figure 8:**
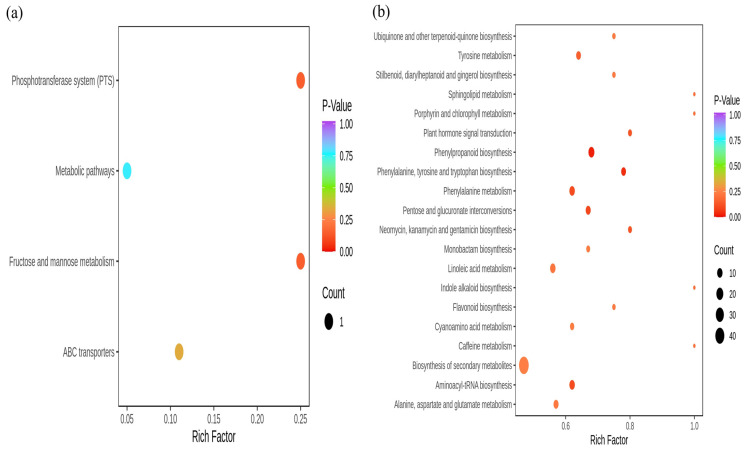
Significant differences in metabolite KEGG pathways in different periods. (**a**): soil; (**b**): leaves. The figure used the *p*-value test.

The differential metabolites screened from the two periods were mapped to KEGG pathways for annotation and pathway enrichment analysis (Figure 8a,b). Differential metabolites in soil were enriched in four metabolic pathways, of which zero were significantly enriched. Differential metabolites in leaves were enriched to twenty pathways, of which seven pathways were significantly enriched, namely Phenylpropanoid biosynthesis, Phenylalanine, tyrosine and tryptophan biosynthesis, Tyrosine metabolism, plant hormone signal transduction, Phenylalanine metabolism, Pentose and glucuronate interconversions, and Aminoacyl–tRNA biosynthesis.

### 3.9. Correlation Analysis between Root Morphology and Soil Environment

**Figure 9:**
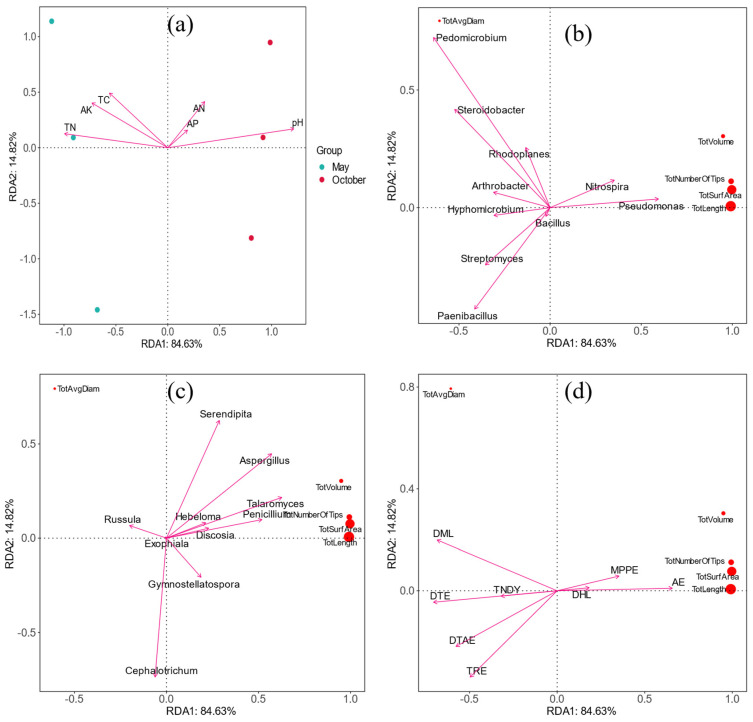
Redundancy of fine root traits and rhizosphere environmental factors. DTAE: D(+)-Talose; TRE: Tridecane; TNDY: 3-Trifluoromethylbenzylamine, N,N-diundecyl; DML: D-Mannitol 2; DTE: D-Turanose; AE: Arabinofuranose; DHL: 1,3-Dipentyl-heptabarbital; MPPE: [2-(6-methyl-2-pyridyl)ethyl](phenyl)-[3-(di-t-butylphosphino)propyl]-Phosphine. Abbreviations—Tot Length: Total root length; Tot Surf Area: Total surface area; Tot Avg Diam: Total the average root diameter; Tot Volume: total volume; Tot Number Of Tips: Total number of the root tips. (**a**) The RDA results for the nutrients and root traits; (**b**) The RDA results for the bacteria and root traits; (**c**) The results of the RDA for fungi and root traits; (**d**) The results of the RDA for differential metabolites and root traits.

In order to explore the effect of the rhizosphere environment on the fine root traits, a redundancy analysis (RDA) was carried out on the rhizosphere nutrients, the top ten bacterial genera and fungi genera, and significantly different metabolites and fine root traits. The RDA results for the nutrients and root traits showed that (Figure 9a) the interpretation rates of the first and second sorting axes for the changes in fine root traits were 84.63% and 14.82%, and the total interpretation rate was 99.54%. Total nitrogen and pH had a significant impact on the fine root traits. The RDA results for the bacteria and root traits showed that (Figure 9b) the interpretation rates of the first and second sorting axes for the changes in fine root traits were 84.63% and 14.82%, and the total interpretation rate was 99.54%. The *Pedomicrobium* and *Steroidobacter* had a significant impact on the fine root traits. The results of the RDA for fungi and root traits showed (Figure 9c) that the interpretation rates of the first and second sorting axes for the changes in fine root traits were 76.42% and 20.97%, and the total interpretation rate was 97.39%. The *Serendipita* and *Cephalotrichum* had a significant impact on the fine root traits. The results of the RDA for differential metabolites and root traits showed that (Figure 9d) the interpretation rates of the first and second sorting axes for the changes in fine root traits were 84.63% and 14.82%, and the total interpretation rate was 99.54%. D-Mannitol 2 and Arabinofuranose had a significant impact on the fine root traits.

**Figure 10:**
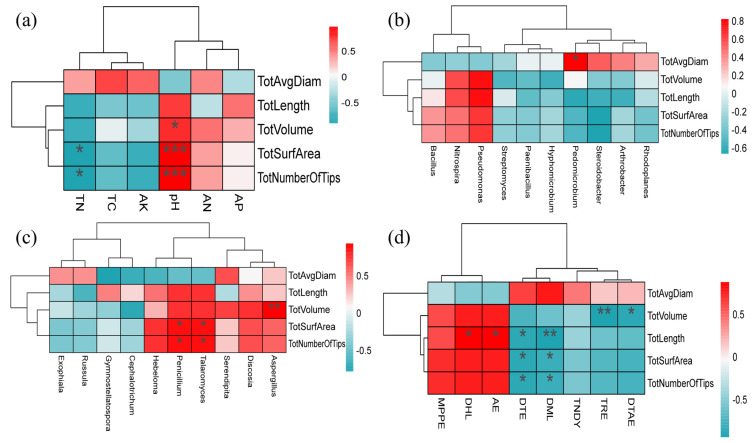
Correlation between fine root traits and rhizosphere environmental factors. Red indicates a positive correlation, blue indicates a negative correlation, and 0.5 indicates a significant correlation. One asterisk means significant (*p* < 0.05), two asterisks mean highly significant (*p* < 0.01), and three asterisks mean very significant (*p* < 0.001). DTAE: D(+)-Talose; TRE: Tridecane; TNDY: 3-Trifluoromethylbenzylamine, N,N-diundecyl; DML: D-Mannitol 2; DTE: D-Turanose; AE: Arabinofuranose; DHL: 1,3-Dipentyl-heptabarbital; MPPE: [2-(6-methyl-2-pyridyl)ethyl](phenyl)-[3-(di-t-butylphosphino)propyl]-Phosphine. Abbreviations—Tot Length: Total root length; Tot Surf Area: Total surface area; Tot Avg Diam: Total the average root diameter; Tot Volume: total volume; Tot Number Of Tips: Total number of the root tips. The single asterisk (*) represents the significance of *p* < 0.05, the double asterisk (**) represents *p* < 0.01, and the triple asterisk (***) represents *p* < 0.001 value. (**a**) The correlation analysis results between the rhizosphere nutrients and the fine root traits; (**b**) The results of the correlation analysis between rhizosphere bacteria and fine root traits; (**c**) The results of the correlation analysis between rhizosphere fungi and fine root traits; (**d**) The results of correlation analysis between rhizosphere metabolites and fine root traits.

The Spearman correlation heatmap assessed the correlation between rhizosphere environmental factors and fine root traits. The correlation analysis results between the rhizosphere nutrients and the fine root traits showed that (Figure 10a) total nitrogen was significantly (*p* < 0.05) negatively correlated with total number of tips and total surface area. Soil pH was significantly positively correlated with the total number of tips and total surface area and significantly (*p* < 0.05) correlated with the total volume of fine roots. In general, the soil’s available potassium, total carbon, and total nitrogen were negatively correlated with fine root traits, while pH, available nitrogen, and available phosphorus were positively correlated with fine root traits. The results of the correlation analysis between rhizosphere bacteria and fine root traits are shown in Figure 10b. The total average diameter was significantly positively correlated with *Pedomicrobium* (*p* < 0.05). Overall, rhizosphere bacteria have more negative correlations with fine root traits. The results of the correlation analysis between rhizosphere fungi and fine root traits are shown in Figure 10c. The Aspergillus was significantly positively correlated with total volume (*p* < 0.05); *Talaromyces* and *Penicillium* were significantly positively correlated with total surface area and total number of tips (*p* < 0.05). Overall, rhizosphere fungi have more positive correlations with fine root traits. The results of correlation analysis between rhizosphere metabolites and fine root traits are shown in Figure 10d. The 1,3-Dipentyl-heptabarbital and Arabinofuranose were each significantly positively correlated with total length (*p* < 0.05). Tridecane, D(+)-Talose was significantly negatively correlated with total volume (*p* < 0.05). D-Turanose and D-Mannitol 2 were significantly negatively correlated with the total number of tips, total surface area, and total length (*p* < 0.05/0.01).

### 3.10. Metabolome and Microbial Association Analysis

**Figure 11:**
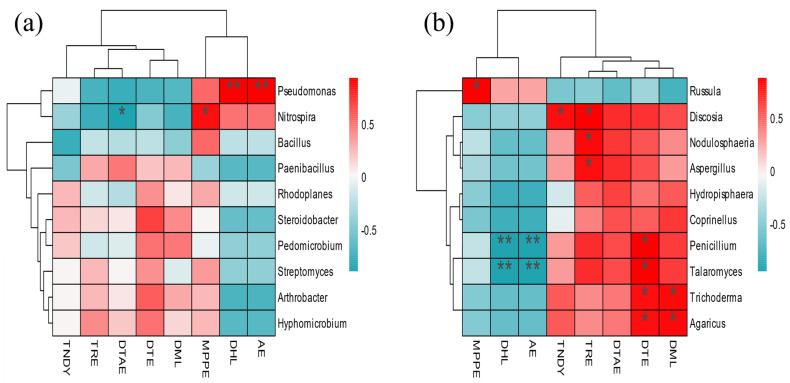
Association of soil microorganisms and metabolome (**a**) bacteria, (**b**) fungi. The single asterisk (*) represents the significance of *p* < 0.05, and the double asterisk (**) represents *p* < 0.01.

In the soil bacteria (Figure 11a,b), *Pseudomonas* was significantly positively correlated with Arabinofuranose and 3-Dipentyl-heptabarbital; *Nitrospira* was significantly positively correlated with 2-(6-methyl-2-pyridyl) ethyl] (phenyl)-[3-(di-t-butylphosphino) propyl]-Phosphine, and significantly negatively correlated with D(+)-Talose. In the soil fungi, seven microorganisms, such as *Discosia*, *Againcus,* and *Trichoderma,* were positively correlated with metabolites such as D(+)-Talose, D-Mannitol 2, and D-Turanose, and negatively correlated with metabolites such as 1,3-Dipentyl-heptabarbital, while *Russula* was the opposite.

**Figure 12:**
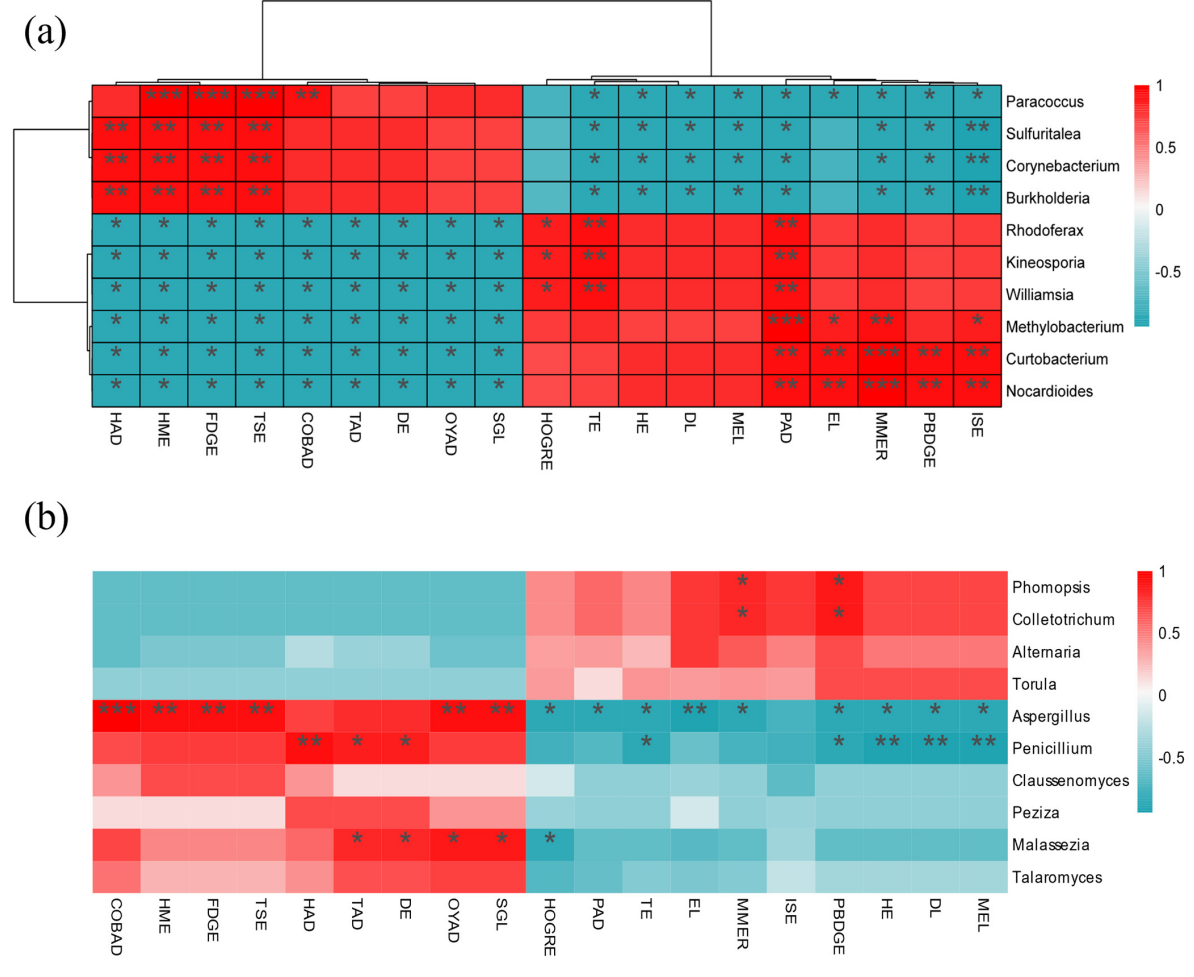
Association analysis of leaf microorganisms and metabolome (**a**) bacteria, (**b**) fungi. MEL: 2-Methoxy-4-ethenylphenol; ISE: Idesin salicylate; HOGRE: 2-Hydroxyphenol-1-O-glucosyl(6→1)rhamnoside; LMER: L-Methionine methyl ester; HE: 8-Hydroxyguanosine; TE: Thymine; FDGE: Furanofructosyl-α-D-(6-mustard acyl)glucoside; COBAD: 3-[(1-Carboxyvinyl)oxy]benzoic acid; TAD: 2,3,4-Trihydroxybenzoic acid; DE: 2′-Deoxycytidine; TSE: Tartronate semialdehyde; HME: 4-Hydroxy-3-methoxymandelate; HAD:β-Hydroxyisovaleric acid; OYAD: 9,12-Octadecadien-6-Ynoic Acid; DGL:1-Stearidonoyl-Glycerol. The single asterisk (*) represents the significance of *p* < 0.05, the double asterisk (**) represents *p* < 0.01, and the triple asterisk (***) represents *p* < 0.001 value.

In the leaf bacteria (Figure 12a,b), *Paracoccus*, *Sulfuritalea*, *Corynebacterium*, and *Burkholderia* were positively correlated with the up-regulation of differential metabolites and negatively correlated with the down-regulation of metabolites; other fungi such as Rhodoferax and Kineosporia were the opposite. In the leaf fungi, *Phomopsis*, *Colletotrichum*, *Altermaria,* and *Torula* were positively correlated with the up-regulation of differential metabolites and negatively correlated with the down-regulation of metabolites. In the leaf bacteria (Figure 12a), Paracoccus, Sulfuritalea, Corynebacterium, and Burkholderia were positively correlated with the up-regulation of differential metabolites and negatively correlated with the down-regulation of metabolites; however, other bacteria such as Rhodoferax and Kineosporia were the opposite. In the leaf fungi (Figure 12b), Phomopsis, Colletotrichum, Altermaria, and Torula were positively correlated with the up-regulation of differential metabolites and negatively correlated with the down-regulation of metabolites.

## 4. Discussion

### 4.1. Root Characteristics and Leaf–Soil Nutrients

The ability of a plant to grow depends largely on the ability of the root system to obtain nutrients and water from the soil. Plant roots are direct sensors of soil environmental changes. In order to adapt to environmental changes, the roots will adopt various strategies to obtain the substances needed for growth. This adjustment is mainly achieved by changing the root traits [29]. In this study, the root system of *I. polycarpa* changed over time, and the range of changes in various indicators was relatively large. The specific performance was that when the root system partially died, the root system grew thicker, and when the number of roots increased, the root system grew. It shows that the root system of young trees is greatly affected by the environment, and environmental changes prompt the root system of young trees to produce new roots and speed up the process of old root death, which is consistent with the research of Bai et al. [30] on the root system of *Populus euphratica* Oliv.

Nutrients are the material basis for plant growth and development. For example, carbon is the basic component of cells, and nitrogen, phosphorus, and potassium participate in the nutrient cycle between plants and the external environment [31]. Soil is a loose layer of matter on the earth’s surface, composed of minerals, organic matter, water, air, etc. These substances constitute the natural medium for microorganisms. Usually, hundreds of millions of microbial cells are found in one gram of soil [32]. In this study, soil total carbon, available nitrogen, available phosphorus, and available potassium showed a trend of increasing and decreasing with the change in time, indicating differences in the accumulation of nutrients in different periods. These elements accumulated more in May and less in October, consistent with Chai’s research results [33] on fragrant pears. The total nitrogen content of soil decreased gradually with the change in growth period, and the soil total nitrogen content of young trees in October was significantly lower than in May. The elemental characteristics of plant leaves are closely related to plants’ basic behavior and function, and the content of each nutrient can reflect the nutritional level of plants [34]. The pH of the soil was above 7 in different periods, indicating that the plants were suitable to be grown in weakly alkaline conditions. Plant leaf carbon and nitrogen elements are crucial in leaf growth [35]. The contents of TC and TN in leaves and soil first increased and then decreased with the growth progress, reaching the maximum value in July, indicating that a large amount of energy needed to be accumulated in July to supply their growth and development. The phosphorus content in leaves is closely related to the available phosphorus content in soil [36]. In this paper, the phosphorus content in soil and leaves was relatively low in May and October, indicating that the phosphorus content in the soil of the experimental woodland in this growth stage was insufficient for seedlings to absorb, and phosphorus fertilizer should be increased in time.

### 4.2. Leaf-Soil Microbial and Metabolome

The microbial community is an important part of the plant micro-ecosystem, and its structure and function are easily affected by various factors such as season, tree species, and living parts. The microbial diversity of rhizosphere soil and leaves varied significantly in this study. There are two reasons for this phenomenon. The first is that the different microorganisms have the most suitable ecological conditions for their growth. The climate in the experimental area is warm and humid in May, and the temperature in October is relatively lower and the area is drier, which affects the activity of microorganisms. Second, the internal physiological activities of plants change with the process of growth and development, affecting the distribution and accumulation of substances in various tissue areas of the tree [37]. These substances cause differences in microbial diversity in different periods and species. Further species classification annotations revealed differences in the main components of the microbial communities in different periods and parts of the plants, and the relative abundance of different species of bacteria and fungi changed taxonomically. The rhizosphere soil bacterial community mainly comprises *Pseudomonadota*, *Acidobacterium*, and *Actinomycetes*, which are enriched in the rhizosphere environment and are related to their environmental solid adaptability [38]. *Pedomicrobium* and *Nitrospira* are both bacteria related to nitrogen metabolism. *Pedomicrobium* belongs to denitrifying bacteria, which can cause soil nitrogen loss. *Nitrospira* belongs to nitrifying bacteria, and nitrification is closely related to soil nitrogen mineralization and nitrogen retention [39,40]. It has been shown that the rhizosphere soil microbial community will adjust according to the soil nutrient status to maintain the ecosystem nutrient balance. The dominant bacteria in rhizosphere soil mainly belong to Ascomycetes and Basidiomycetes. The dominant bacteria in the rhizosphere in May contain a variety of beneficial bacteria and some pathogens, such as *Basketomycetes* [41] and *Mortierella* [42], which play a role in promoting plant mineral absorption, the decomposition of plant residues, and disease resistance, while *Alternaria* [43] is the pathogen causing plant diseases.

The dominant bacterial phyla in leaves were mainly *Pseudomonadota*, consistent with the results of Liu et al. [44]. From May to October, the number of dominant bacterial genera in leaves increased, which may be related to the changes in intrinsic material composition in leaves at different stages of development. The dominant flora of leaf fungi are similar to the rhizosphere soil region, and Ascomycota is the main dominant phyla in the two regions. However, compared with leaves, the rhizosphere soil fungal flora has more species, which is consistent with the conclusion of Ren et al. [45] on bayberry. At the genus level, the dominant fungi in May were more abundant than in October. Among the dominant bacteria in the May leaves, *DOK59* is closely related to the denitrification process of N_2_O being reduced to N_2_ [46]. *Burkholderia* can promote plant growth and development, improve nutrient absorption, and increase stress resistance [47]. The dominant bacteria in leaves in October, *Bacillus*, also has a significant relationship with plant growth and development [48]. *Discosia* [49], *Alternaria* [50], and *Phomopsis* [51] in leaf fungi are pathogenic in some studies and are the main pathogens that cause plant diseases. Previous studies have found that summer is the main period when Phyllostachys chinensis is prone to disease, and the damage may continue until winter [52]. The results of this paper also show that young *Phyllostachys chinensis* trees are infected by pathogens.

Based on GC-MS technology, eight differential metabolites were screened out in the soil of the two periods, of which four were up-regulated, and four were down-regulated, including five types of substances such as carbohydrates, other types, alcohols, heterocyclic compounds, and lipids. The differential metabolites in the soil are mainly carbohydrates. Carbohydrates are the main energy substances used for cell metabolism and the main carbon source for microorganisms [53]. In this study, the relative content of carbohydrates in the rhizosphere soil in May was significantly higher than in October. Using UPLC-MS/MS technology to analyze the leaves in the two periods, 302 differential metabolites were screened out, which can be attributed to phenolic acids, amino acids, and their derivatives, nucleotides and their derivatives, organic acids, lipids, and other categories. Among the ten metabolites with the most significant differences, seven out of the ten compounds with the largest up-regulation were phenolic acids; seven out of the ten with the largest down-regulation were phenolic acids.

### 4.3. Association of Dominant Microorganisms with Root Morphology, Nutrients, and Differential Metabolites

The relationship between nutrient factors and the dominant bacteria and fungi was analyzed, and it was found that nutrient status impacted the soil and leaf microbial communities. The correlations between bacteria, fungi, and nutrient factors differed in different tissue areas. The soil bacterial community was significantly correlated with soil pH, available potassium, phosphorus, and total nitrogen. The main factors influencing the soil bacterial communities in different periods were significantly different. Most of the dominant bacteria in the rhizosphere soil in May were positively correlated with the soil’s available potassium and total nitrogen. Most of the dominant bacteria in the soil in October were positively correlated with available phosphorus and pH. The positive correlation may be due to the fact that, in different growth and development periods, the roots release relevant exudates into the soil, which changes the pH and nutrients of the rhizosphere soil environment and causes different types of bacteria accumulation. The soil pH and available potassium are the main factors controlling the soil fungal communities in the rhizosphere. The research of Zhou [54] found that soil pH is a key driver of microbial community structure, which can indirectly or directly regulate the number of soil microorganisms in the rhizosphere of plants. The significant correlation between the rhizosphere soil fungal community and soil available potassium is consistent with the results obtained by Liao et al. [55]. Our study combined results for the soil bacteria and fungi in the rhizosphere and found that some beneficial microorganisms in the soil, such as *Skermanella* [56], which can resist heavy metal stress, and *Arthrobacter*, *Rhizomycetes*, *Streptomyces*, and *Penicillium*, *Trichoderma*, *Aspergillus*, etc., which have biological control functions, are positively correlated with soil available potassium and total nitrogen. The copper *greedy*, *pseudomonas,* and *rhizobium*, which are positively correlated with soil pH, are beneficial to soil nutrient fixation and have growth-promoting effects on plant growth [57,58].

Nutrient factors affected the leaf microbial community more than the soil microbial community. Leaf total carbon, phosphorus, and total potassium were significantly correlated with the leaf bacterial and fungal communities, while total carbon and total nitrogen significantly affected the leaf fungal communities. Leaf total potassium significantly impacted beneficial microorganisms such as *Pseudomonas*, *Burkholderia*, *Aspergillus,* and *Trichoderma*. Gui et al. [59] found that *Burkholderia* contains some potassium-decomposing bacteria, which can decompose some potassium-containing minerals and release potassium elements that plants can directly utilize. Luo et al. [60] also isolated and identified a *Pseudomonas* with high potassium-solubilizing activity. *Sphingomonas* is the dominant flora in many plants. Many studies have reported the functions of *sphingomonas* in degrading toxic substances, resisting pathogenic bacteria [61], and promoting plant growth [62]. In this study, leaf pH is the main factor influencing *Sphingomonas*. *Penicillium*, as the main antagonistic flora in the endophytic fungi on the leaves of *P. chinensis*, is closely related to total carbon, nitrogen, and total potassium in leaves. The above results show that soil pH, available potassium, total nitrogen, and leaf pH, total carbon, total nitrogen, and total potassium are important driving factors affecting the microbial community and play an active role in the microecological regulation of rhizosphere soil and leaves. Among them, rhizosphere nutrients are substances that can be directly utilized by the root system and are closely related to the growth of the root system.

In this work, RDA showed that pH, *Pedomicrobium*, *Talaromyces*, *Penicillium*, and D-Mannitol 2 in rhizosphere soil significantly influenced roots. As a signal, pH activates the cell wall porosity and elongation process [63], which can affect plant growth and development by promoting cell wall extension. In this paper, the pH of the rhizosphere was positively correlated with fine root traits, which also verifies that *I. polycarpa* is suitable for growth under weakly alkaline conditions [64]. The Pedomicrobium is a typical Mn (II)-oxidizing bacterium. It changes the pH of the soil through its growth and development and participates in the recycling and utilization of Mn in the soil in synergy with other microorganisms [65]. It also plays a vital role in removing environmental metal ion pollution, degrading soil organic matter, and inhibiting the growth of pathogenic bacteria [66]. The *Talaromyces* can improve plant resistance to environmental stress and nutrient absorption by promoting the absorption of nutrients (such as nitrogen, phosphorus, and trace elements) from biological fertilizers and control agents [67]. *Penicillium* can promote growth and improve plant disease resistance [68]. In our study, they are positively correlated with root traits, indicating that they are promoting the growth of the root system. Through analysis, this study found that the metabolites in the rhizosphere have more negative effects on the fine roots. Some studies have found that organic matter in the rhizosphere soil of plants has a toxic effect on plants [69]; for example vanillic acid, D-mannitol-2, β-sitosterol, and carotene in the continuous cropping of *Rehmannia glutinosa* have different inhibitory effects on the growth, and similar substances may also exist in the root metabolites.

The plant microorganisms form a special micro-ecological environment, and a stable ecological cycle is carried out between the two [70]. Microorganisms use organic compounds such as plant root exudates and metabolites to increase their proliferation. At the same time, the products of organic matter decomposition and metabolism by microorganisms, in turn, affect the growth of plants. Sugar is an important carbon and energy source for microorganisms. In the rhizosphere soil dominant bacteria, the dominant soil bacteria showed different degrees of correlation with carbohydrate metabolites such as D-Turanose and 3-Trifluoromethylbenzylamine. The heat map showed that the correlation between the bacterial community and differential metabolites was opposite to that of the fungal community, which may be related to the different functions played by bacteria and fungi in leaf physiological and metabolic activities. There are 10 substances that had a significant positive correlation with the leaf bacterial community, and the types of substances were relatively complex, including lipids, phenolic acids, organic acids, amino acids and their derivatives, and nucleotides and their derivatives, indicating that bacteria play a significant role in regulating leaf biological functions. The leaf fungal community was positively correlated with 10 substances, such as phenolic glycosides and catechols. The types of these 10 substances were mainly phenolic acids and organic acids. Some phenolic and organic acids in metabolites may change the leaf microenvironment (such as pH, moisture, etc.) [71], indirectly affecting the composition of the leaf fungal community.

## 5. Conclusions

The root system’s growth is mainly based on the increase in fine roots and root elongation, which is conducive to nutrient absorption. During the growth period, there are differences in the utilization of soil total nitrogen, leaf pH, total nitrogen, total phosphorus, and total potassium by young *Idesia polycarpa* trees. The diversity of microorganisms in the soil and leaf groups changed, and the relative abundance of soil-dominant bacteria in young trees decreased. The dominant bacteria in young trees changed from Proteus to *Pachydermis*, and phenolic acids were the crucial differential metabolites in the growth period of young trees. The root morphology of young trees is mainly affected by pH, *Pedomicrobium*, *Talaromyces*, *Penicillium*, and D-Mannitol 2. However, based on the complexity of the plant microecological environment, the study of the main influencing factors of the microecology in this paper is only in the preliminary exploration stage, and further exploration research can be carried out in the future.

## Data Availability

The data presented in this study are available on demand from the first and correspondence authors.
